# Supplementary material for: Interplay between topological valley and quantum Hall edge transport
Source: Nat Commun. 2022 Jul 20;13:4187. doi: 10.1038/s41467-022-31680-y (PMC9300606; doi:10.1038/s41467-022-31680-y)
Supplement: Supplementary file 1 — Supplementary Information [file 41467_2022_31680_MOESM1_ESM.pdf]

Supplementary Information for

Interplay between topological valley and quantum Hall edge transport

Fabian R. Geisenhof[1], Felix Winterer[1], Anna M. Seiler[1],
Jakob Lenz[1], Ivar Martin[3], R. Thomas Weitz[1,2,4,5]*

Affiliations:

[1]Physics of Nanosystems, Department of Physics, Ludwig-Maximilians-Universität München, Geschwister-Scholl-Platz 1, Munich 80539, Germany

[2]1st Physical Institute, Faculty of Physics, University of Göttingen, Friedrich-Hund-Platz 1, Göttingen 37077, Germany

[3]Materials Science Division, Argonne National Laboratory, Lemont, Illinois 60439, USA

[4]Center for Nanoscience (CeNS), LMU Munich, Schellingstrasse 4, Munich 80799, Germany

[5]Munich Center for Quantum Science and Technology (MCQST), Schellingstrasse 4, Munich 80799, Germany

*Corresponding author. Email: thomas.weitz@uni-goettingen.de

Supplementary Note 1:

Identifying the emerging broken-symmetry quantum Hall states in the presence of a stacking domain wall

Since the conductance of the appearing quantum Hall states in device D1-DW differs quite significantly from usually observed values as in device D2, we have additionally recorded fan diagrams to examine the slope of transconductance fluctuations[1,2] (see Supplementary Fig. 1). As some of the emerging phases show an electric field dependence, we have measured the conductance at various applied fields (see Supplementary Fig. 1a – d). The slopes of the appearing broken-symmetry states fit very well to the expected $\nu = 0, \pm1, \pm2$ and ±4 states, despite all having similar conductances of 3 – 4 $e^2\ h^{-1}$. Thus, additional charge transport along the domain wall in parallel to the quantum Hall edge states is unambiguously the cause for the higher conductance in the different states. Whereas the $\nu = 0$ phase is most stable for low and high electric field, consistent with a phase transition from the LAF/CAF to the LP phase[3,4], the $\nu = \pm4$ is most stable for low electric field. Contrarily, the (partially) layer polarised $\nu = \pm1, \pm2$ phases appear only at intermediate electric field at these low magnetic field. Notably, since at $n = E = B = 0$ transconductance fluctuations with zero slope are visible, the layer antiferromagnetic phase is indeed present but masked owing to the quantum valley transport along the stacking domain wall.

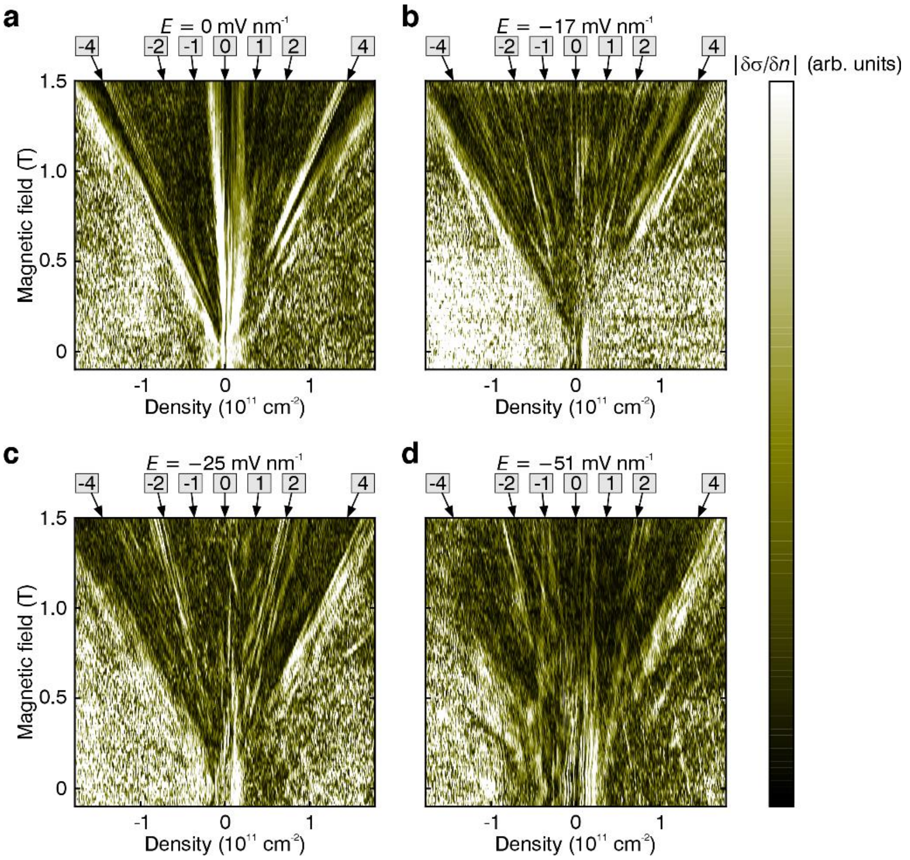

Supplementary Figure 1 | Fan diagrams measured at specific electric fields. a – d, Derivative of the differential conductance $\delta\sigma/\delta n$ as a function of magnetic field and charge carrier density for various electric fields. The slopes of the individual broken-symmetry $\nu = 0, \pm1, \pm2, \pm4$ states are indicated with arrows.

Supplementary Note 2:

Phase transition between the fully layer polarised and the canted antiferromagnetic $\boldsymbol{\nu = 0}$ phase in the presence of a stacking domain wall

When sweeping E as a function of B for zero charge carrier density (Supplementary Fig. 2a,b), the transition between the fully layer polarised and the canted antiferromagnetic phase appears as region with increased conductance in both devices, consistent with previous measurements[3,4]. However, in device D2 both phases are insulating, whereas in D1-DW the conductance remains finite at $\sigma \approx 2.9\, e^2\, h^{-1}$ (see Supplementary Fig. 2c) due to the kink states contributing to the charge transport. Within the CAF phase the conductance is slightly decreasing for increasing magnetic field (Supplementary Fig. 2d), consistent with the increase of canting and evolving energetic dispersion of the kink states (see main text). In Supplementary Fig. 2e, the band structure of the CAF and LP phase in the presence of a domain wall are schematically shown. In contrast to the minigap opening in the CAF phase owing to the hybridising of same valley states, the valley-helical kink states in the LP phase remain largely intact due to the suppression of intervalley scattering.

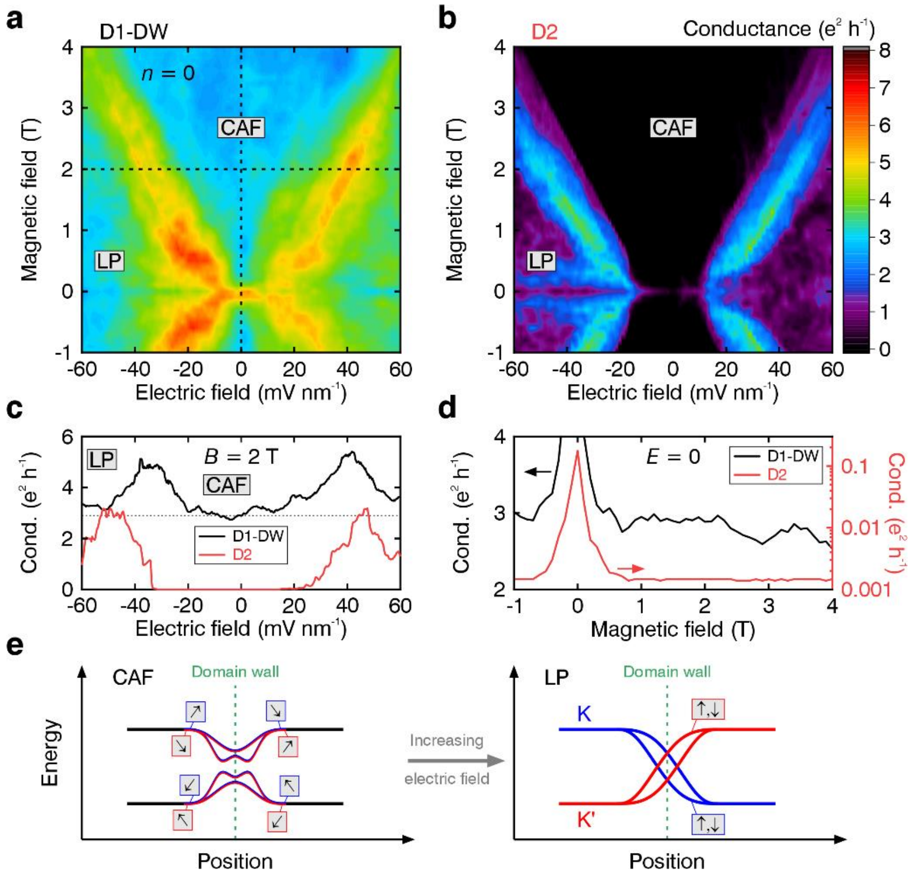

Supplementary Figure 2 | Phase transition between the fully layer polarised and the canted antiferromagnetic $\nu = 0$ phase. a,b, Conductance as a function of applied electric *E* and magnetic field *B* at zero charge carrier density for device D1-DW (a) and D2 (b). The first-order phase transition between the two $\nu = 0$ phases, the CAF and the LP phase, is characterized by a region with increased conductance. The dashed lines in (a) indicate the position of the data shown in (c) and (d). **c,** Line traces of the conductance across the $\nu = 0$ phase transition shown for sample D1-DW (black) and D2 (red) at *B* = 2 T. The dashed line marks the value $2.9\ e^2\ h^{-1}$. **d,** Conductance as a function of *B* for *n* = *E* = 0. The data of device D1-DW (D2) is shown in black (red). **e,** Schematic band structure as a function of position around a stacking domain wall shown for the CAF and LP phase.

Supplementary Note 3:

Impact of domain walls on the band structure beyond the zero energy Landau level

Supplementary Fig. 3 shows the influence of the domain wall on higher Landau levels. To this end, we have recorded a conductance map as a function of function of E and n at B = 1.5 T, see Supplementary Fig. 3a. At this low magnetic field, we can observe the $\nu = \pm 8, \pm 12$ quantum Hall states. While the conductance of the $\nu = \pm 8$ states show the expected conductance, the one of the $\nu = \pm 12$ state seems to be lower than 12 $e^2\ h^{-1}$. Moreover, we see an oscillating behaviour of the conductance when entering a new quantum Hall plateau (see Supplementary Fig. 3a,b). One possible explanation for these oscillations could be the rather low aspect ratio $L/W \approx 0.5$ of the device, with L and W being the length and width of the device channel, respectively. It has been shown that the shape of a sample can non-trivially affect the conductance at the quantum Hall transitions[5].

However, theoretical calculations of Landau levels energies (see Supplementary Fig. 3c,d) show that domain walls can cause the formation of ripples within higher Landau levels. For the band structure calculation, we used a linearized model of graphene layers near K/K' valley, with smoothly varying interlayer hybridisation across the domain wall; namely, hopping A1B2 gradually is being replaced by B1A2. The energies are calculated in a finite width strip, with the domain wall in the middle. In Landau gauge, the translational invariance in the direction of the strip is preserved, and the energies are plotted as a function of momentum along the strip. The rescaled momentum also corresponds to the locations x of the centres of individual states, when rescaled by $c/(eB)$, with c being the speed of light. Interestingly, if the expected increase of A1A2 and B1B2 hybridisation at the domain wall is not included, the zero energy Landau levels remain flat through the domain wall. In contrast, the higher Landau levels show

significant variations – “ripples” – near the domain wall. This could also be the possible reason for the observed oscillations of conductance as a function of density.

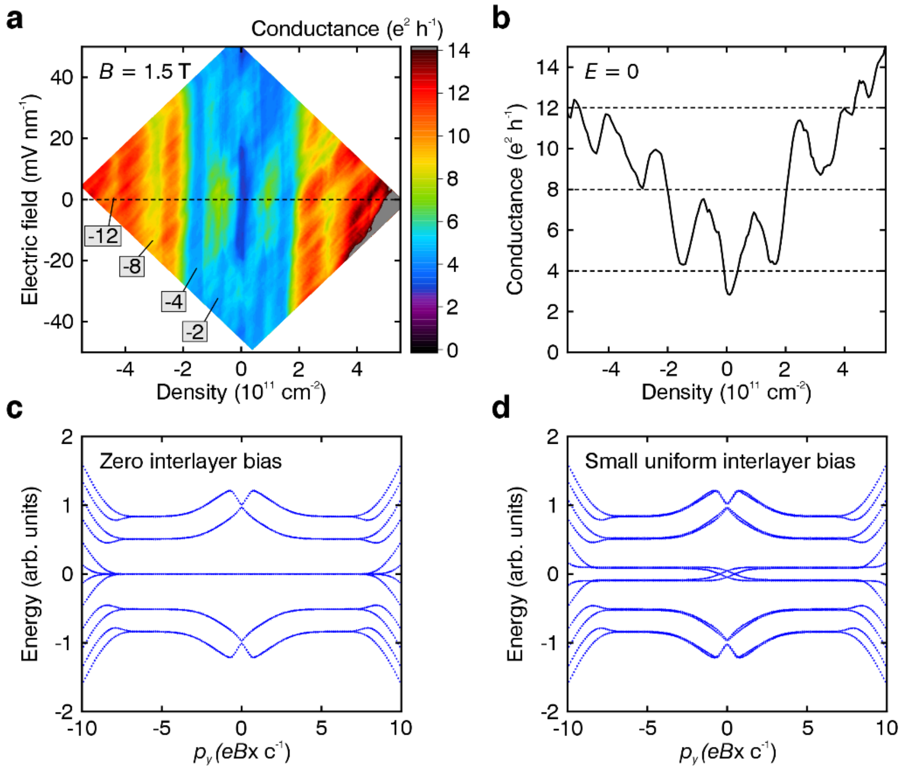

Supplementary Figure 3 | Influence of the domain wall on the energetic landscape of higher Landau levels. a, Conductance as a function of E and n at B = 1.5 T. The dashed line indicates the position of the data shown in (b). **b,** Line trace of the conductance as a function of charge carrier density for zero electric field. The dashed lines indicate multiples of $4\, e^2\, h^{-1}$. **c,** Schematic Landau level band structure computed in the presence of a domain wall, smoothly interpolating between AB and BA stacking. The horizontal axis is the momentum along the domain wall. In Landau gauge used here, it is proportional to the location of the centre of a given orbital, x. With zero interlayer bias, the zeroth Landau level is four-fold degenerate (valley and orbital index), and higher Landau levels are doubly degenerate owing to valley degeneracy (spin is ignored). Near the edges the Landau levels float away from zero energy. The behaviour near the domain wall ($x = 0$) depends on the precise way that domain wall interpolates between AB and BA stackings. Even when the zeroth Landau level is flat (when A1A2 and B1B2 hopping near domain wall is ignored), the higher Landau levels are sensitive to the presence of the stacking defect. **d,** same as in (c) but with a small uniform interlayer bias. Notably, valley-helical modes emerge at the domain wall.

Supplementary Note 4:

Full quantum transport data at low and high magnetic field in the presence of a stacking domain wall

Supplementary Fig. 4a – e shows the full conductance maps as a function of E and n for various magnetic fields in device D1-DW. Most prominently, the spectral minigap emerges for $B \geq 8$ T causing the conductance to drop, marked by the cross in Supplementary Fig. 4c – e and in the line traces in Supplementary Fig. 4f – h. Additionally, the conductances of the $\nu = 0, \pm 1, \pm 2$ states are dropping for increasing magnetic field. This can also be observed in the line traces shown in Supplementary Fig. 4f – h. For the $\nu = 0$ CAF phase, the decrease can be explained by the emergence of a minigap due to the hybridising of partially spin aligned counterpropagating modes in the same valley, as explained in the main manuscript. For the $\nu = 0, \pm 1, \pm 2$ states, we think the effect occurs owing to increased intervalley scattering, as explained in the main manuscript.

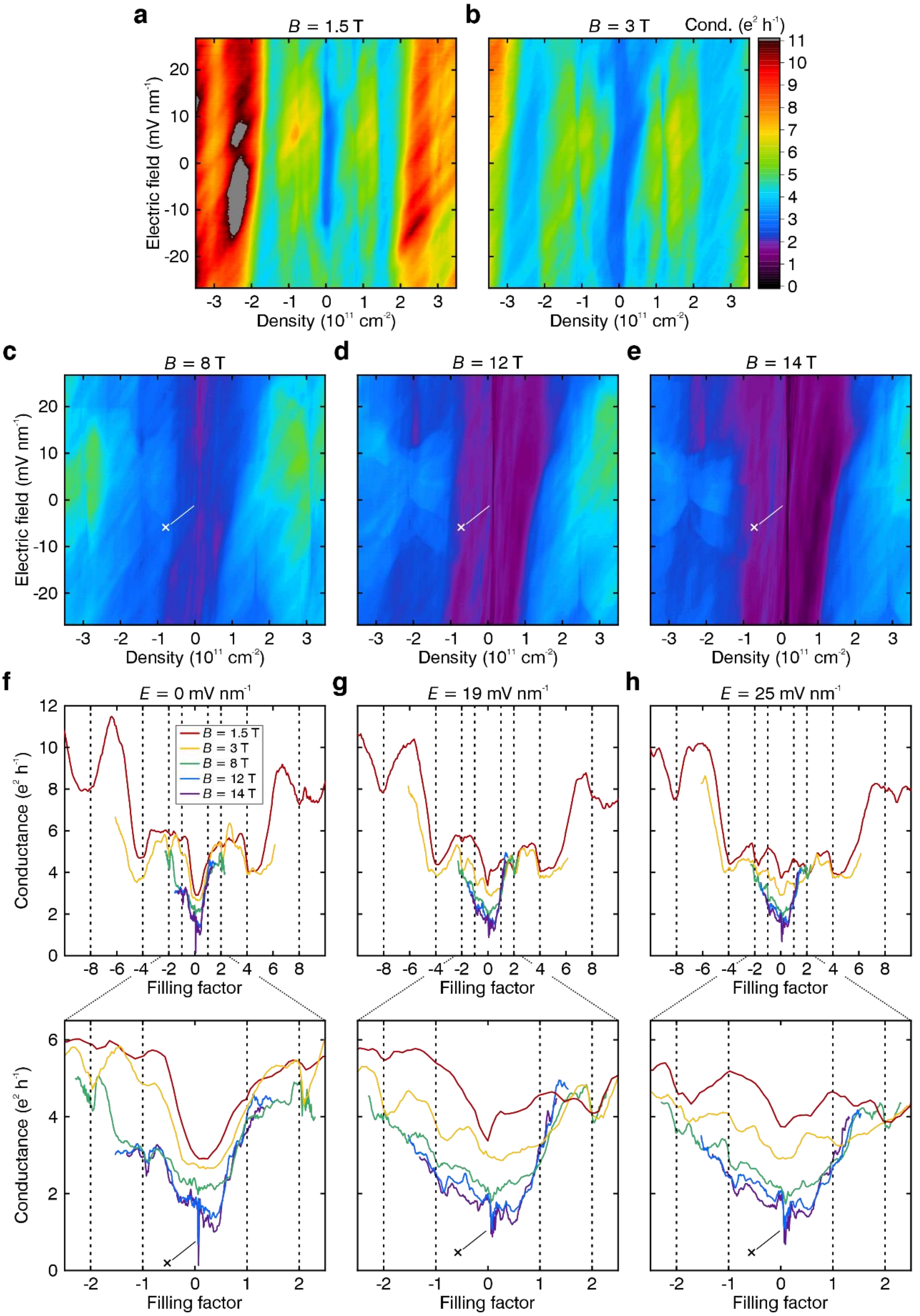

Supplementary Figure 4 | Topological valley and quantum Hall edge transport for low and high magnetic fields. a – e, Maps of the conductance as a function of electric field and charge carrier density for various magnetic fields in device D1-DW. The cross indicates the conductance dip caused by the appearing minigap. **f – h,** Line traces of the conductance as a

function of filling factor for E = 0, 19 mV nm^{-1} and 25 mV nm^{-1}. The lower panels are zoom-ins around small filling factors.

Supplementary Note 5:

Persistence of the spectral minigap for a different cooldown

Supplementary Fig. 5a shows a fan diagram recorded as a function of back gate voltage in device D1-DW. The graph shows the emergence of the spectral minigap within the $\nu = 0$ phase at $B \geq 8$ T, indicated by the cross. Moreover, its evolution for increasing magnetic field can be seen in Supplementary Fig. 5b, which shows line traces of the conductance for various high magnetic fields. The dip in conductance is increasing for increasing magnetic field, which matches the observations shown in Fig. 3 in the main manuscript.

It is worth noting that the data shown in Supplementary Fig. 5 was recorded during a different cooldown of the device than the data shown in the main manuscript. Most importantly, this demonstrates the persistence of the spectral minigap over multiple cooldowns. Since each cooldown involves a current annealing procedure, driving high currents through the device seem also to not affect the emergence of the feature. Notably, the device D1-DW was not as clean during the cooldown corresponding to Supplementary Fig. 5 as for the measurements shown in the main manuscript (in terms of residual charge disorder and contact resistance), which makes the direct comparison of absolute values of the conductance difficult.

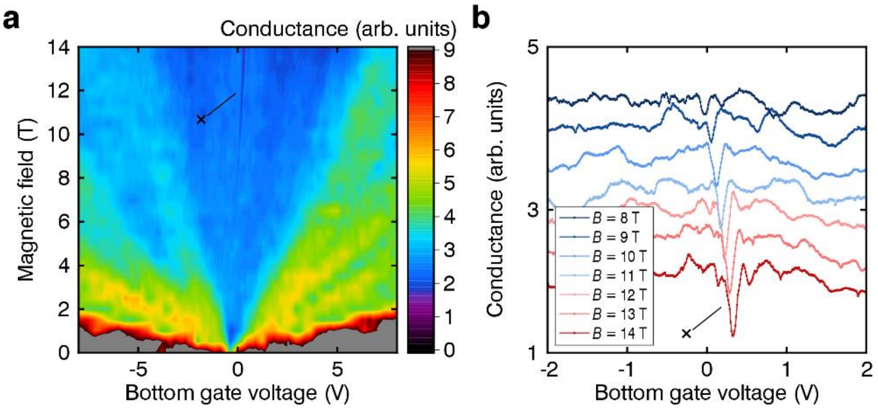

Supplementary Figure 5 | The emergence of a spectral minigap within the $\nu = 0$ phase for high magnetic field. a, Fan diagram showing the conductance as a function of magnetic field and bottom gate voltage. The cross indicates the conductance dip caused by the emergence of the spectral minigap. Note that the data was recorded with device D1-DW but during a different cooldown than the measurements shown in the main manuscript. **b,** Line traces of the conductance as a function of back gate voltage at various magnetic fields. The data is taken from the fan diagram shown in (a). The line cuts are offset for better visibility.

Supplementary Note 6:

Quantum transport measured in additional devices

Supplementary Fig. 6 shows the data from three additional devices with domain wall. Supplementary Fig. 6a – c shows the conductance of the $\nu = 0, -1, -2, -4$ quantum Hall states as a function of magnetic field measured in the devices D2-DW, D3-DW and D4-DW, respectively. Note that the conductance was averaged over the regime at which the respective state emerges, i.e. for the $\nu = 0$ CAF phase around zero electric field, for the $\nu = -1$ and -2 at $|E| \geq 10$ mV nm^{-1} and $|E| \geq 15$ mV nm^{-1} and the $\nu = -4$ state at all electric fields. The full conductance maps as a function of E and n for various magnetic fields are shown in Supplementary Fig. 6d – f for the three devices.

Similar to device D1-DW, all three samples show a decrease of the conductance for the quantum Hall states with increasing magnetic field. Although in device D2-DW (Supplementary Fig. 6a) and D3-DW (Supplementary Fig. 6b) the decrease is very prominent, sample D4-DW (Supplementary Fig. 6c) shows only a slight decrease of conductance. Moreover, a clear minigap can only be observed in device D2-DW (see Supplementary Fig. 6a,d). However, we have indications that the quality of the devices D3-DW and D4-DW is significantly lower than that of D2-DW or even D1-DW. In device D4-DW, even at low magnetic field, the conductances of the $\nu = 0, -2, -4$ states differ greatly. Evidently, the additional conductance originating from the kink states is highly reduced due to a low quality of the domain wall. In device D3-DW, the CAF phase can only be clearly observed for $B \geq 3$ T. Both observations indicate an overall lower quality of the two devices. Hence, the minigap can probably not be resolved due to disorder in both samples.

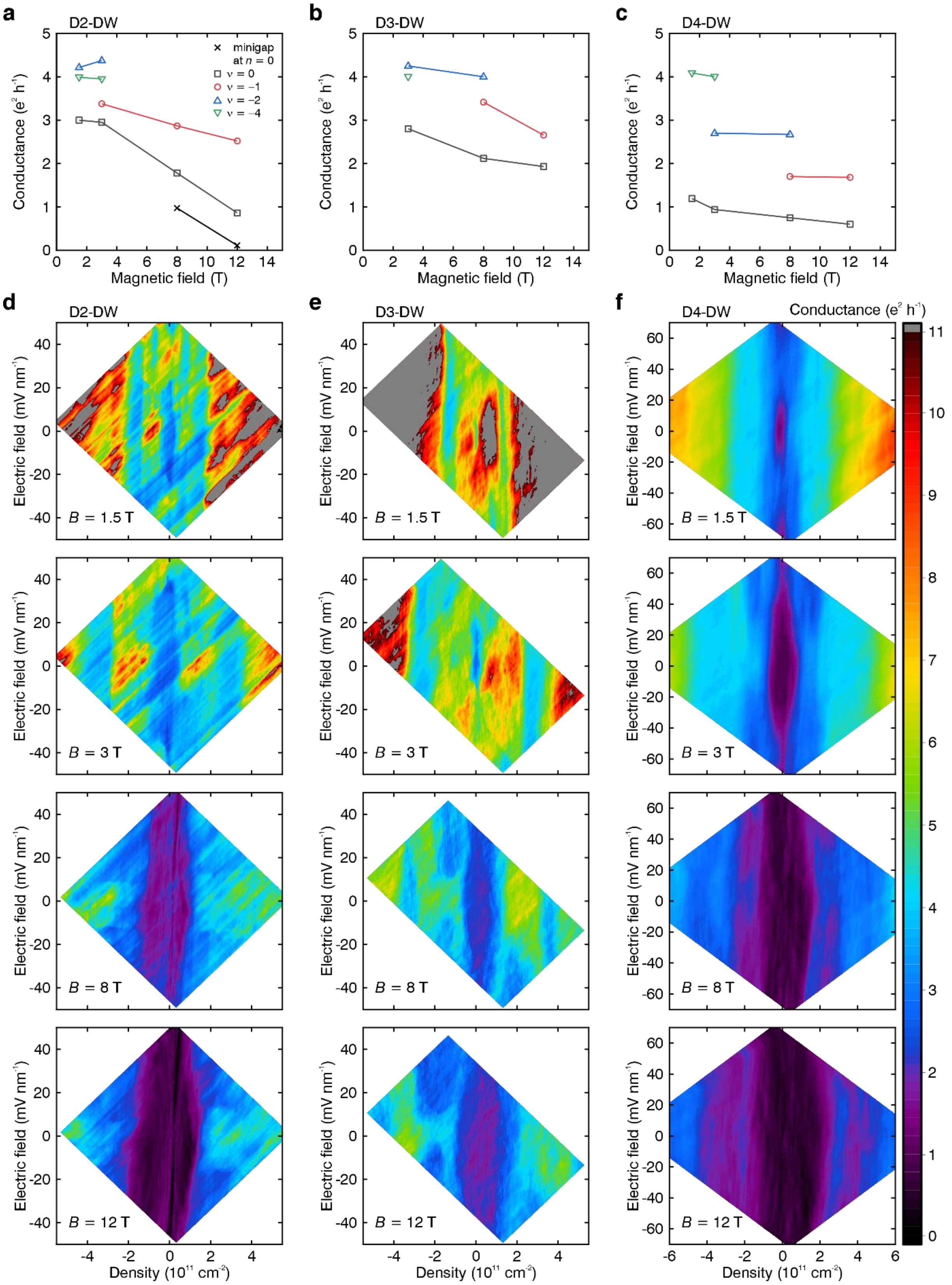

Supplementary Figure 6 | Data from additional devices. a – c, Conductance of the $\nu = 0, -1, -2, -4$ QH states as a function of B for the devices D2-DW (a), D3-DW (b) and D4-DW

(c). For device D2-DW, also the conductance within the spectral minigap is shown. **d – f,** Maps of the conductance as a function of E and n for various magnetic fields for the three devices.

References

1. Lee, D. S., Skákalová, V., Weitz, R. T., Klitzing, K. von & Smet, J. H. Transconductance fluctuations as a probe for interaction-induced quantum Hall states in graphene. *Phys. Rev. Lett.* **109**, 56602 (2012).
2. Geisenhof, F. R. *et al.* Quantum anomalous Hall octet driven by orbital magnetism in bilayer graphene. *Nature* **598**, 53–58 (2021).
3. Weitz, R. T., Allen, M. T., Feldman, B. E., Martin, J. & Yacoby, A. Broken-Symmetry States in Doubly Gated Suspended Bilayer Graphene. *Science* **330**, 812–816 (2010).
4. Maher, P. *et al.* Evidence for a spin phase transition at charge neutrality in bilayer graphene. *Nat. Phys.* **9**, 154–158 (2013).
5. Abanin, D. A. & Levitov, L. S. Conformal invariance and shape-dependent conductance of graphene samples. *Phys. Rev. B* **78**, 035416 (2008).
